# An EANM position paper on advancing radiobiology for shaping the future of nuclear medicine

**DOI:** 10.1007/s00259-022-05934-2

**Published:** 2022-09-06

**Authors:** EANM Radiobiology Working Group:, Jean-Pierre Pouget, Mark Konijnenberg, Uta Eberlein, Gerhard Glatting, Pablo Minguez Gabina, Ken Herrmann, Søren Holm, Lidia Strigari, Fijs W. B. van Leeuwen, Michael Lassmann

**Affiliations:** 1 Institut de Recherche en Cancérologie de Montpellier (IRCM), INSERM U1194, Université de Montpellier, Institut Régional du Cancer de Montpellier (ICM), 208 Rue des Apothicaires, 34298 Montpellier, France; 2 Radiology & Nuclear Medicine Department, Erasmus MC, Rotterdam, The Netherlands; 3 Department of Nuclear Medicine, University of Würzburg, Würzburg, Germany; 4 Department of Nuclear Medicine, Ulm University, Ulm, Germany; 5 Department of Medical Physics and Radiation Protection, Gurutzeta-Cruces University Hospital/Biocruces Health Research Institute, Barakaldo, Spain; 6 Department of Nuclear Medicine, University of Duisburg-Essen and German Cancer Consortium (DKTK)-University Hospital Essen, Essen, Germany; 7 Department of Nuclear Medicine, Rigshospitalet, University Hospital Copenhagen, Copenhagen, Denmark; 8 Department of Medical Physics, IRCCS Azienda Ospedaliero-Universitaria Di Bologna, Bologna, Italy; 9 Interventional Molecular Imaging Laboratory, Department of Radiology, Leiden University Medical Center, Leiden, The Netherlands

## Introduction

Personalized medicine is an important facet of medical radiation research programs and roadmaps of strategic research agendas (EURADOS or EURAMED) [1, 2]. Indeed, patient-specific dosimetry in radiopharmaceutical therapy (RPT) and particularly for treatment planning is progressively implemented in clinical practice (ICRU report 96 and ICRP Publication 140 [3, 4]). However, we often still lack radiobiological understanding to address the clinical unmet needs (EANM radiobiology position paper [5] and [6–12]). Selection of patients for RPT based on imaging confirmation of the RPT-directed target alone seems to be insufficient as even in the VISION clinical trial more than half of the PSMA-PET positive patients showed a PSA-decrease of less than 50% [13]. In RPT, most treatments are administered systemically and in addition to tumor tissue some part of the body may receive high absorbed doses. However, RPT is generally well tolerated and the current therapy regimens seem to undertreat patients which again strongly underlines the need for a better radiobiological understanding of the required tumor doses, the radiation tolerance of the organs at risk as well as the potential synergistic value of combination therapy. Because radiobiology of EBRT cannot be extrapolated to RPT, there is a need for specific radiobiology of RPT [6].

The EANM radiobiology working group, therefore, intends to promote and foster the advancement of research in radiobiology stimulating the collection, analysis, and understanding of radiobiological data specific for RPT. An increased radiobiological understanding will lead to direct benefits for patients and improve treatment outcomes by also complementing the clinical development of new RPT concepts. Main areas of radiobiological research include high level biological studies for the identification of rational combination-partners for RPT, the establishment of RPT-specific normal organ absorbed dose limits, as well as the intensified treatment schedules exploiting the full therapeutic index of RPT [14].

## Suggested research topics

To advance radiobiological insights, the prospective collection of more preclinical and clinical data is urgently needed. The complementary value of preclinical and clinical radiobiology research projects will help rationalize the therapeutic use of radiopharmaceuticals. Hereby the aim is to come up with evidence-based treatment planning (injected activities, treatment duration, and fractionation), balance therapeutic efficacy and reducing potential toxicities, and exploring synergism of combination therapies. The following objectives are all part of this ambition.

### Objective 1: large patient datasets available for radiobiology projects

Although increasing, the number of patients receiving RPT is still quite low representing often heterogeneous patient cohorts. Moreover, when RPT is not used as 1st line of treatment the time between de novo diagnosis and RPT can be long and it is preceded by first- and second-line therapies.

Typically RPT is given post NETTER-1 in 2nd line in well-differentiated neuroendocrine tumors (after somatostatin analogs) [15] and post VISION directly after 1 line of novel anti-androgen receptor-directed therapies and 1 line of taxane-chemotherapy in mCRPC [13].

This leads to a large data variability and complicates the interpretation of pure radiobiological effects. To overcome this barrier, we need to come up with means to gather patient information at different stages of therapy (before-, during-, and after RPT). Such data must include standard clinical parameters such as patient’s age, sex, tumor type, tumor grade, previous treatments, lifestyle, and environmental factors. At the same time tumor samples and liquid biopsies must be collected prior and/or after RPT and registered in biobanks according to standardized procedures. At last, the collection of data on RECIST and PERCIST criteria used to assess the patient response as well as the standardized uptake values for PET scans and dosimetry data is of great importance. Toxicities and other possible late effects (e.g., myelodysplasia) also seem critical and need to be correctly reported.

The goal of objective 1 is to provide the European Nuclear Medicine scientific community with a comprehensive set of standardized data and patient samples that comply with the FAIR (Findability, Accessibility, Interoperability, and Reuse of digital assets) principles. The theranostic center initiative recently put forward by the EANM [16, 17] could help gathering comprehensive data sets of standardized patient data for radiobiological research. Through artificial intelligence methods that identify potential causal associations, FAIR database can help identify parameters among large cohorts of patients that modulate RPT efficacy and toxicity and help investigate the dose–effect relationship.

The European Health Data Space, aiming at facilitating sharing of health data, across Europe, should develop clear interoperability targets and incentives for data collection and generation, especially for procedures with small cohorts of patients. This is to be hoped that the European Health Data Space, leveraging the examples of the cancer registries, will facilitate data collection and data analysis. Similarly, the EU action of standardization of electronic health records should consider appropriate ways of integrating information on patient radiation absorbed dose.

### Objective 2: relevant preclinical models for radiobiology

To fill in the gaps in the clinical findings, preclinical models of RPT need to be developed. Models should closely mimic clinical situations in a controlled environment. Objective 2 includes the development of biological models including 2D and 3D in vitro cultures of murine and human cancer cells, organoids, and genetically modified animal models for in vivo studies. The tissues considered are the tumor and its microenvironment (e.g., immune and endothelial cells, fibroblasts, adipocytes) and healthy tissues (e.g., bone marrow, liver, kidney, salivary glands). Similar to the clinical data collection, it is important to standardize techniques and data collection, preferably in a way that allows preclinical and clinical data to be related to each other.

### Objective 3: recommendations on dosimetry in preclinical models

Used as a referential, the absorbed dose assessment is a pre-requisite for radiobiological studies because it allows comparing different targeting situations. Objective 1 will provide information on patient dosimetry. In objective 3, we propose to give recommendations on dosimetry assessment in preclinical models. Small-scale dosimetry models have been developed for cells, animals, and patients to take into account the non-uniform uptake in relation to the heterogeneous exposure of functional units in the elements under study and related to the biological end-points. The activity uptake input data for these models should comply with quality standards that need to be defined. Minimal requirements to define cellular uptake kinetics should be described and should include data on specific cell compartments and cell morphology. Guidance documents on biodistribution approaches for dosimetry assessment are needed to allow traceable comparisons among studies.

Preclinical dosimetry models for in vitro (cell) and in vivo (animal) models strongly depend on the radiation transport mechanisms used in these models. A common radiation transport model needs to be established to solve possible dosimetry outcome discrepancies in preclinical studies. Reporting of dosimetry, dose-related effects, and radiobiological end-points should follow a common nomenclature to increase the overall standards. These standards are certainly needed when combination therapies are tested to determine the presence of synergetic effects and the possible risks of increased toxicity.

### Objective 4: tumor and normal tissue radiobiology

As mentioned in the “Introduction” section, RPT radiobiology cannot be reliably extrapolated directly from EBRT. RPT differs from EBRT in terms of absorbed dose, dose rate, exposure protraction, dose distribution, the potential biological activity of the targeting molecule, and use of radionuclides that emit various particles (beta, alpha, Auger, gamma/X) and of low and high LET radiation mixtures. Objective 4 is about investigating the biological mechanisms involved in RPT. Besides the DNA radiobiology measured in targeted cells or non-targeted cells (formation, repair, signaling pathways associated with the DNA damage response in vitro and in vivo, genomic instability), RPT physical features highlight the need to consider specifically also non-DNA-centered effects, particularly the cell membrane, mitochondria, lysosomes, and endoplasmic reticulum. Biological effects at short (bystander) or long distance from the irradiated cells, in cells not targeted by the radiopharmaceuticals, also need to be investigated. This requires considering intercellular communications between tumor cells and healthy cells and/or cells in the tumor microenvironment (e.g., endothelial cells, cancer-associated fibroblasts, immune cells) and extracellular matrix. Therefore, it is an integrated radiobiology approach at the molecular/cellular and tissue scale that needs to be developed in relevant preclinical models and also in patients. Absorbed dose assessment (described in objective 3) is a pre-requisite to tumor and normal tissue radiobiology.

### Objective 5: standardized protocols for preclinical and clinical radiobiology

Standard assays and techniques should be used in preclinical models and up to patient samples by all research teams in the field of RPT radiobiology. Therefore, radiobiology (objectives 1 and 4) will benefit from establishing standardized protocols. This will allow data comparison and exchanges among laboratories and hospitals in multi-center trial settings and according to the FAIR principles. Objective 5 includes setting up standardized procedures for establishing biobanks, collecting patient samples and data, data analysis, and also for radiobiological assays. Early-phase clinical trials of new radiopharmaceuticals should follow these standards. Moreover, new designs for absorbed dose escalation steps should be developed based on the acquired knowledge on the dose–effect relations obtained in preclinical RPT studies and the corresponding EBRT absorbed dose limits.

### Objective 6: dose–effect relationship and quantitative radiobiological models

Preclinical models (objective 2) allow exploring the relationship between the absorbed dose delivered to the tissues and RPT biological effects without the limitations imposed by the clinical settings. Accurate dose assessment (objective 3) and data obtained in objective 1 (patients) and objective 2 (preclinical models) will allow investigating the dose–effect relationships and developing tools using established and new quantitative radiobiological models that will consider α, β, µ parameters, biological effective dose (BED), equivalent uniform dose (EUD), and equivalent uniform BED (EUBED).

Besides the calculation of the absorbed dose delivered to tissues, these studies should also investigate how absorbed dose, dose fractionation, and irradiation duration modulate tumor and healthy tissue (toxicities) responses to RPT. Objective 6 will provide information on how low dose rate, repair, and recovery can influence the response to RPT. Dose–response models should take these data into account, together with the immune and bystander effects in low-dose irradiated regions.

### Objective 7: biomarkers of RPT response

Currently, it is challenging to predict RPT efficacy and side effects. Moreover, some radiation-induced effects manifest at later time points. Therefore, it is important to identify early biological markers that can be used in patients. This will be possible only in large-scale studies using patients’ samples that take advantage, for example, of high-throughput OMICs techniques. As imaging techniques and theranostic medicine are at the heart of nuclear medicine, artificial intelligence and radiomics approaches are a major advantage of this field. This objective stresses again the need for large datasets (objective 1).

### Objective 8: optimized RPT and potential combination approaches in RPT

By taking advantage of the findings delivered in the previous objectives, it should be possible to propose personalized and rationalized treatments leading to the highest therapeutic index. The data gathered in the previous objectives will allow determining for each patient the activity to administer, the fraction number, and the interval between fractions. They will also make possible to find the best combination(s) between RPT and immunotherapies, DNA repair inhibitors, immune-response modulators, EBRT, and other treatments still under study.

### Objective 9: regulatory advice/guidance for including radiobiology in clinical trials

Registration dossier includes the description of the pharmacodynamic properties of the radiopharmaceutical under investigation. However, it must be noted that biological activity of radiopharmaceuticals is mainly driven by radiation effects, since the concentration of the (unlabeled) drug used is usually far below the level needed for pharmacological effects. The regulatory requirements for the marketing authorization of radiopharmaceuticals are part of the Samira action plan to identify and find solutions for situations where these regulations conflict with the council directive 2013/59/EURATOM, according to which each radiation therapy should have a patient-specific exposure optimization [18]. However, optimization requires knowledge about how radiation modulates biological effects according to dose–response relationship during RPT, which is not the case to date. Therefore, molecule/radionuclide-related dose–response relationships should be published. Furthermore, more attention should be paid to the clinical endpoints considered by providing available patient data according to objective 1.

To overcome this, clinical radiobiology needs to be considered by investigating in patients the relationship between dose delivered and biological outcomes. It is only possible if clinical trials include access to biological resources by referring to objective 5. This should be part of the radiopharmaceutical registration dossier and should be added to the description of the pharmacodynamic properties of the radiopharmaceutical under investigation.

### Objective 10: multidisciplinary teaching

Education of nuclear medicine physicians, biologists, physicists, and other researchers is needed to develop and implement patient-specific theranostic options. A better understanding of radiobiology should be incorporated into the training curriculums of all specialities involved. Another potential option for teaching radiobiology for nuclear medicine could be to establish specialized course in the framework of the European School of Multimodality Imaging & Therapy (ESMIT, https://www.eanm.org/esmit/about-2/), which represents the EANM’s response to massive changes in the educational needs of the nuclear medicine community and the rising demand for greater multimodality content.

## Conclusion

In conclusion, there is a tremendous need to better understand the radiobiology of RPT improving patient care, patient survival, and innovation of new RPT concepts. In addition to the urgent requirement of prospective large data collection, there is the demand of a global and integrated approach to the study and understanding of the biological effects of ionizing radiation in the context of RPT. The outlined ten objectives document how the massive development of clinical and preclinical radiobiological approaches can be achieved with the new wave of theranostic centers potentially playing a pivotal role.
